# Attention U-net for automated pulmonary fissure integrity analysis in lung computed tomography images

**DOI:** 10.1038/s41598-023-41322-y

**Published:** 2023-08-29

**Authors:** Zachary W. Althof, Sarah E. Gerard, Ali Eskandari, Mauricio S. Galizia, Eric A. Hoffman, Joseph M. Reinhardt

**Affiliations:** 1 5601 Seamans Center for the Engineering Arts and Sciences, University of Iowa Roy J. Carver Department of Biomedical Engineering, Iowa City, IA 52242 USA; 2 University of Iowa Department of Radiology, Iowa City, IA USA; 3 University of Michigan Department of Radiology, Ann Arbor, MI USA

**Keywords:** Biomedical engineering, Respiration, Predictive markers

## Abstract

Computed Tomography (CT) imaging is routinely used for imaging of the lungs. Deep learning can effectively automate complex and laborious tasks in medical imaging. In this work, a deep learning technique is utilized to assess lobar fissure completeness (also known as fissure integrity) from pulmonary CT images. The human lungs are divided into five separate lobes, divided by the lobar fissures. Fissure integrity assessment is important to endobronchial valve treatment screening. Fissure integrity is known to be a biomarker of collateral ventilation between lobes impacting the efficacy of valves designed to block airflow to diseased lung regions. Fissure integrity is also likely to impact lobar sliding which has recently been shown to affect lung biomechanics. Further widescale study of fissure integrity’s impact on disease susceptibility and progression requires rapid, reproducible, and noninvasive fissure integrity assessment. In this paper we describe IntegrityNet, an attention U-Net based automatic fissure integrity analysis tool. IntegrityNet is able to predict fissure integrity with an accuracy of 95.8%, 96.1%, and 89.8% for left oblique, right oblique, and right horizontal fissures, compared to manual analysis on a dataset of 82 subjects. We also show that our method is robust to COPD severity and reproducible across subject scans acquired at different time points.

## Introduction

**Figure 1:**
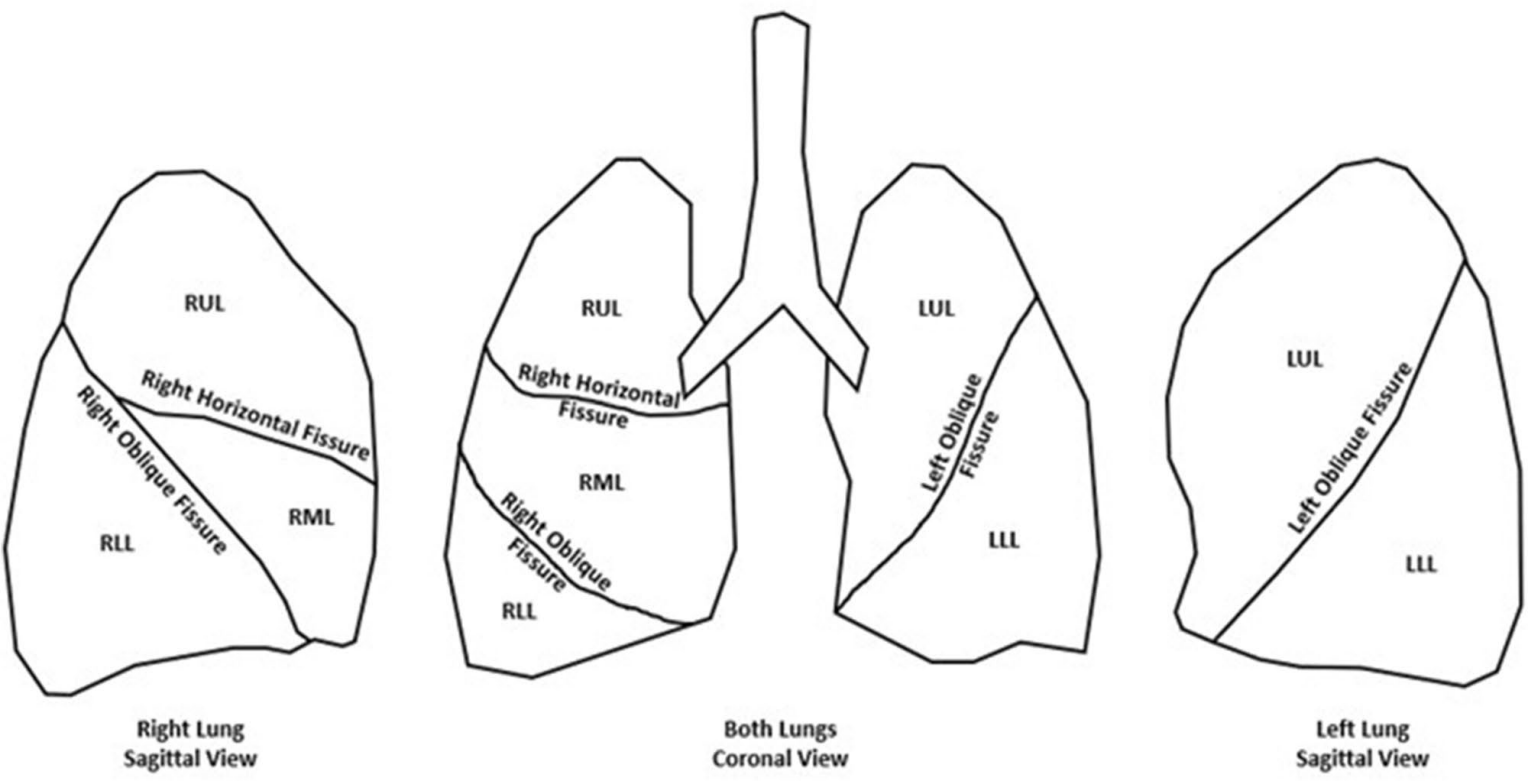
Fissures of the lungs. The right horizontal fissure separates the right upper lobe (RUL) and middle lobe (RML). The right oblique fissure divides the middle lobe (RML) and the lower lobe (RLL) anteriorly, and the RUL and RLL posteriorly. The left oblique fissure separates the left upper lobe (LUL) and lower lobe (LLL).

Pulmonary fissures have recently gained attention due to their implications in endobronchial valve treatment screening and lobar sliding^1–5^. The lungs are divided into lobes by invaginations of visceral pleural surface called fissures (see Fig. 1). Fissures can completely or partially separate the lobes from each other. Incomplete separation of pulmonary lobes has been shown to be a biomarker of collateral ventilation between lobes which reduces the efficacy of treatments (e.g., endobronchial valves) that aim to improve lung function by blocking airflow to diseased lung regions^1–4^. Complete fissures may also allow lobes to slide past each other during respiration which has been shown to affect lung biomechanics^5^.

Pulmonary fissure completeness, or integrity, varies greatly between individuals^6–9^. A study visually examining 250 high-resolution computed tomography (CT) images reported that the left oblique fissure was incomplete in 24% of subjects, while 35% of right oblique fissures were incomplete, and 74% of right horizontal fissures showed incompleteness^6^. In a separate study, fifty lungs from cadavers were examined for variations in fissure completeness showed the oblique fissure was incomplete in over 30% of right lungs and in over 50% of the left lungs examined^8^.

Visual assessment of pulmonary fissures from CT images can be a time-consuming and arduous process given the potential for hundreds of image slices to be analyzed. A high degree of reader experience is required to correctly identify fissure integrity from CT images, and it has been demonstrated that inter-reader variability is statistically significant^10^. Rapid, reproduceable, and non-invasive collateral ventilation screening requires automatic fissure integrity assessment from medical images. Automatic fissure integrity analysis is also needed for widescale analysis of lung biomechanics and disease in large datasets. Previous methods have been developed to perform evaluation of pulmonary fissure integrity^10–13^. The method in^11^ utilizes a five-step pipeline to detect fissures, estimate complete/incomplete lobar boundary regions, and estimate fissure integrity using overlap between detected fissure and the estimated complete lobar regions. Another study detected incompleteness by identifying regions where the fissure and lobar segmentations did not align and reported areas under the ROC curve above 0.80 for each fissure^10^. A recent study developed an open-source framework to measure the surface area overlap between the lobar boundary surface and the derived fissure mesh surface as an indicator of fissure integrity^12^. The method described in^12^ reported correlation coefficients between visual scores and their approach of 0.542, 0.679, and 0.851 for the left oblique, right oblique, and right horizontal fissures, respectively. In the past year, a patch-based deep learning model was developed to assess fissure integrity^13^.

In this study we present a deep-learning method for whole-image automatic pulmonary fissure integrity analysis. Utilizing a variation of U-Net, a network architecture that has shown promising results in imaging segmentation tasks, we show that our method can accurately and quickly assess the extent of fissure completeness by classifying voxels on the predicted lobar boundary as intact or incomplete fissure.

## Methods

**Figure 2:**
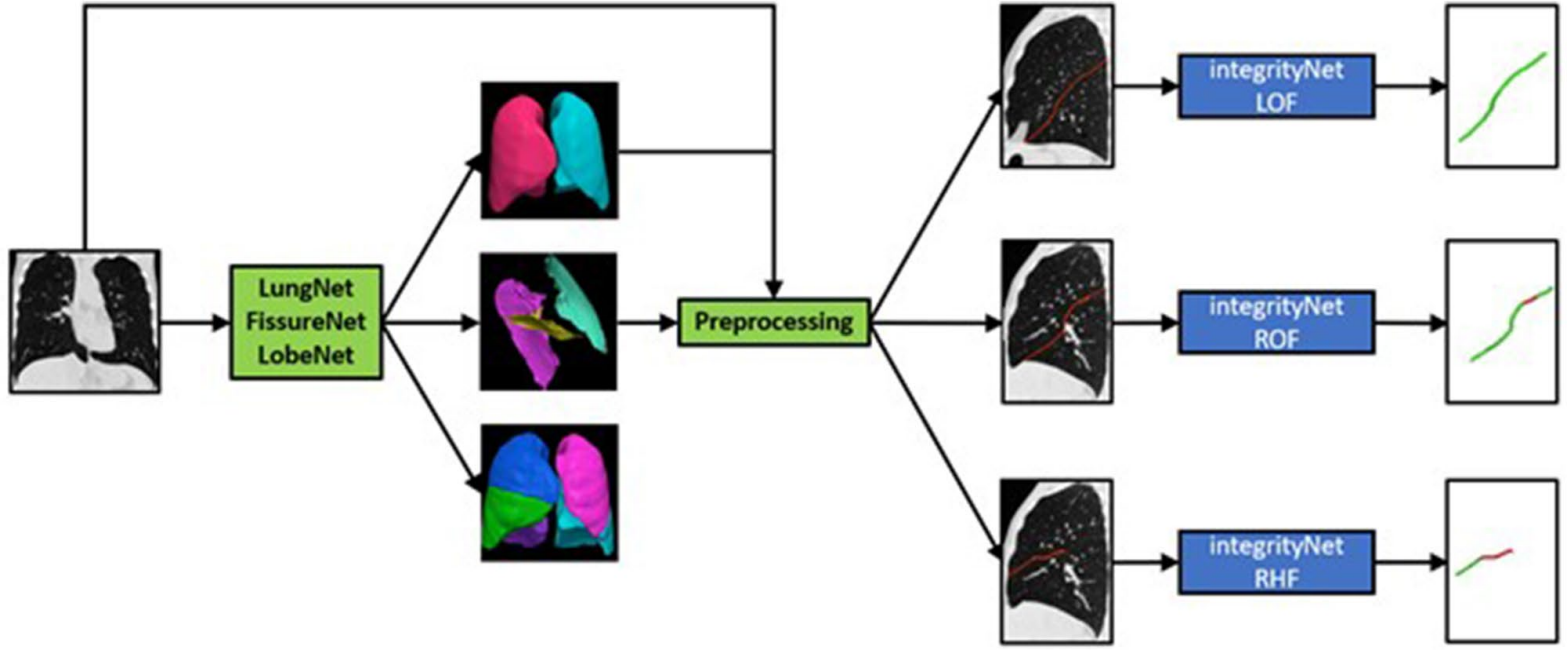
IntegrityNet pipeline. A CT image is input to the LungNet–FissureNet–LobeNet pipeline to obtain segmentations or the lungs, fissures, and lobes. During preprocessing the CT intensity values are clipped to (−1024, 200) and then linearly rescaled to (−1, 1). Next, the lung mask is used to compute a bounding box for each of the left and right lungs. The bounding boxes are used to crop the CT image and the fissure probability image to the left and right lungs. Lastly, the cropped CT and fissure probability images are concatenated along the channel dimension and the result is used as input to IntegrityNet. The output of each IntegrityNet is a fissure integrity mask where green and red represents intact and incomplete fissure, respectively.

In this section, we outline the data used in this study and the ground truth labeling process used to generate training data for our method as well as the pipeline we propose for automatic fissure integrity assessment. Figure 2 depicts the IntegrityNet pipeline described below.

### Imaging Data

The SubPopulations and InteRmediate Outcome Measures in COPD Study (SPIROMICS) was the source of the 3D CT images used to train and test the model proposed in this work. SPIROMICS is a multicenter (including 12 US-based university clinics) and prospective cohort study of subjects with chronic obstructive pulmonary disease (COPD) and non-smoking controls^14^. This study was approved by the institutional review board at the University of Iowa (IRB-01). The SPIROMICS protocol was approved by the IRBs of all participating institutions (Columbia University IRB 2, University of Iowa IRB-01, Johns Hopkins IRB-5, University of California Los Angeles Medical IRB 1, University of Michigan IRBMED B1 Board, National Jewish Health IRB, University of California San Francisco IRB Parnassus Panel, Temple University IRB A2, University of Alabama at Birmingham IRB #2, University of Illinois IRB #3, University of Utah IRB Panel Review Board 5, Wake Forest University IRB #5, and University of North Carolina at Chapel Hill Non-Biomedical IRB). All procedures were carried out in accordance with relevant guidelines and regulations and written informed consent was provided by all subjects. Participants have CT scans at baseline, two-year, three-year, and five-year follow-ups. For each subject both total lung capacity (TLC) and residual volume (RV) scans were acquired^14^. Further scanning protocol details are described by^15^.

The Global Initiative for Chronic Obstructive Lung Disease (GOLD) system is widely used to classify COPD subjects by disease severity based on spirometric lung function^16^. Measurement of expiratory flow rate and volume by spirometry is used to assign subjects to a GOLD stage. GOLD 1 is mild, GOLD 2 is moderate, GOLD 3 is severe, and GOLD 4 is very severe disease. Additionally, in this study we used GOLD 0 subjects who are asymptomatic, but also have a history of smoking, and subjects with no smoking history labeled as “never smokers.”

This work used baseline TLC scans for network training and testing. The one-year and five-year follow-up scans were used to test the reproducibility of network predictions, with the assumption that fissure integrity would not change during the course of the study. Subjects from each of the six classes (GOLD 0–4, and never smokers) and with baseline, one-year, and five-year follow-up scans were randomly selected, for a total of 108 subjects in the cohort. Twenty-six subjects were omitted from the study due to poor image quality and/or anatomic variation leading to errant fissure segmentations that were not able to be used as inputs to the network. Of the 82 subjects remaining, the number of subjects in each disease group is displayed in Table 1.

**Table 1:** Number of subjects from each disease group used in this study.

Status	Count
Never smokers	16
GOLD 0	14
GOLD 1	15
GOLD 2	12
GOLD 3	13
GOLD 4	12

### FissureNet and LobeNet

Several automated fissure detection methods have been developed; however, a key challenge has been the detection of weak and abnormal fissures and reducing the detection of false positive structures^17–20^. FissureNet is a deep learning framework for detection of fissures in CT images which consists of a cascade of two CNNs^18^. FissureNet has been evaluated on several large datasets and compared to other fissure detection methods it is robust to detecting radiographically weak fissures, blurred or abnormal fissure appearance, poor image quality, and images acquired at expiration (RV and FRC). In the case of missing or incomplete fissures, FissureNet does not necessarily provide the complete information to separate the lungs into lobes. LobeNet was developed to utilize the output of FissureNet and enforce the lungs to be separated into five unique lobes^21^. In this work, IntegrityNet utilizes the fissure confidence from FissureNet, along with the original CT image, to assess fissure integrity.

### Ground truth labeling

Ground truth fissure integrity segmentations were obtained by having a pair of trained image analysts (ZA and AE) hand annotate images overseen by a board-certified chest radiologist (MG). LobeNet (see section "FissureNet and LobeNet") was used to generate lobe segmentations^21^. The complete fissure boundary was extracted from the lobe segmentation by identifying lobe boundary voxels adjacent to another lobe. The result is a complete fissure boundary which includes intact fissure as well as incomplete fissure. The complete fissure segmentation was manually inspected, and all voxels were marked as either intact or incomplete. Manual edits were performed by simultaneously viewing the three imaging planes with open-source 3D Slicer software^22^. One analyst (AE) inspected each subject’s scan carefully and marked challenging cases for radiologist review. The second analyst (ZA) reviewed each ground truth image generated by the first analyst to ensure quality and consistency of edits.

### Preprocessing

The proposed method assumes that each CT image has a corresponding lung segmentation, lobe segmentation, and FissureNet’s confidence information in the form of a fissure probability map. All images were resampled to isotropic voxel size (1 mm^3^). The lung mask was used to crop each image into two separate smaller images representing the images localized to the left and right lung, respectively. This reduces input size which reduces memory use during training as well as removing irrelevant information. The right lung image was used twice for each subject, once for right oblique fissure integrity assessment and once for right horizontal fissure integrity assessment. The CT image is further processed to clip intensities to the range (−1024, 200) HU and then the intensities are linearly scaled between (−1,1).

### IntegrityNet

**Figure 3:**
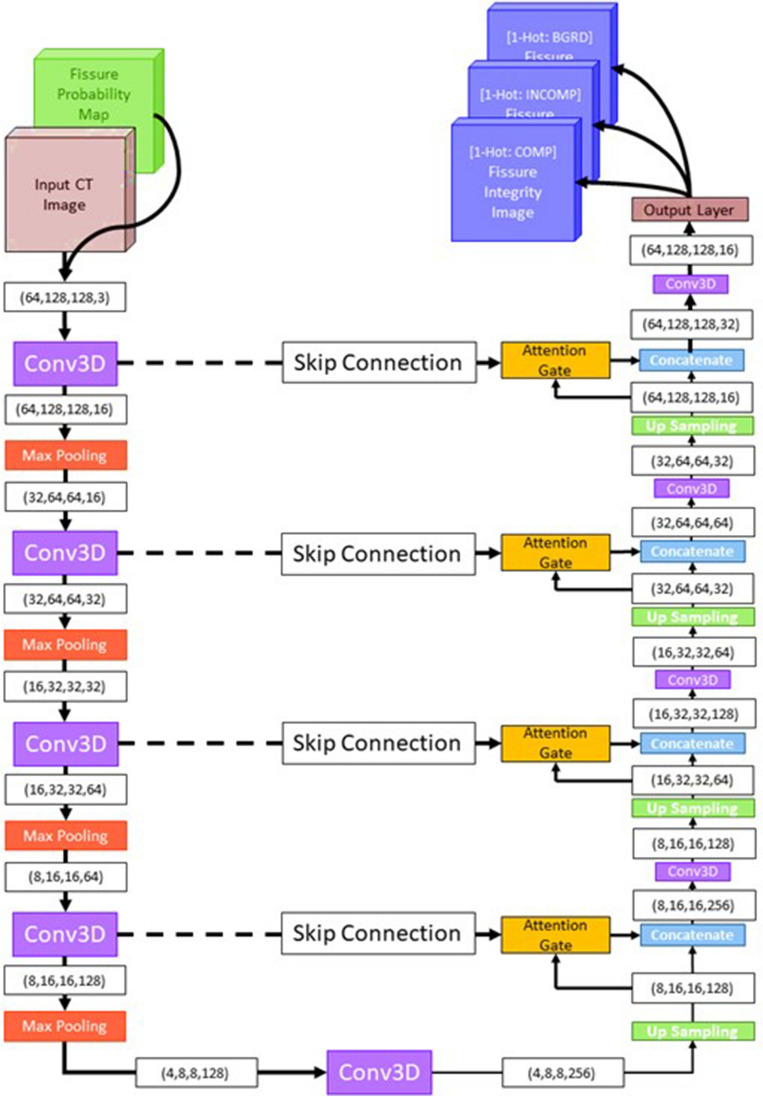
IntegrityNet architecture. The left side of the network represents the contracting path where inputs are progressively down-sampled and features are extracted at each layer. The right side of the network represtents the expanding path where feature maps are progressively up-sampled to generate the final fissure integrity labels. The skip connections bring infromation from the contracting path into the expanding path to improve performance at each layer of up-sampling.

A diagram of IntegrityNet’s architecture can be seen in Fig. 3. The input to the network is the 3D CT image and fissure probability map, concatenated along the channel dimension. The output of the network is a map which indicates the probability of each voxel being visible fissure, incomplete fissure, or background. Spatial dimensions of the input and output are identical. Four encoding steps were used convolving the inputs with a set of kernels to produce increasingly lower resolution/higher order features that describe the input data. The decoding path mirrors the encoding up-sampling the encoded representation of the inputs. Finally, a full-resolution output image is produced identifying regions of visible completeness and incompletes along the fissure. The U-Net structure’s advantage is the skip connections that prevent the loss of high-frequency information by passing information from the encoding path directly to the decoding path^23^. Features are adaptively filtered so that only the most useful information is passed to the expanding path by adding attention gates at skip connections^24^. Three independent networks, each having the proposed IntegrityNet architecture were trained separately on one of the three pulmonary fissures.

### Post-processing

Three post-processing steps are applied to the network output to produce the final fissure integrity image. The first step is to remove false positives from the network prediction. This is done by assigning all voxels outside of the complete fissure to the non-fissure class (background). Next, regions within the complete fissure labeled background are assigned to the majority vote class of its neighbors that were labeled intact or incomplete. This process removes false negatives from the image. Finally, a smoothing operation is performed to remove inconsistent labels. A 26-connectivity neighborhood is used to smooth each voxel along the complete fissure segmentation, where the output label is the majority vote of all of the non-background neighbors.

### Implementation

The IntegrityNet architecture is implemented with the open-source framework Keras^25^. The network was trained using NVIDIA GPU card Tesla P40 with 24 GB RAM. Adam optimization^26^ was used for training with a static learning rate of 0.0002. Tversky loss^27^ was used with α = 0.05 in order to handle the large class imbalance between background and the incomplete and complete fissure classes that lie along the thin fissure surface (i.e., there are many more voxels labeled background in the output image than voxels assigned to intact or incomplete fissure classes).

During training, random cropping to fixed input size of (128, 128, 64) for each image was used to diversify the data seen in training for each epoch. The effect of this method is to increase the amount of data within the training set without the need for more subject images. A validation set was also used to identify the epoch that produced the best results for a set that was held out from training and testing. The train, test, and validation proportions used were 0.75, 0.15, and 0.10 respectively.

## Results

In order to evaluate the IntegrityNet’s performance, k-fold cross validation ($k=8$) was performed to reduce bias in the metrics reported. We examine fissure integrity score error to assess the model’s success in predicting the quantitative representation of the degree of fissure completeness. Fissure integrity percent (FI%) is computed as:

$$FI\mathrm{\%}=(\# voxels\, intact\, fissure/\# voxels\, complete\, fissure)*100.$$

In this way FI% is always a value [0.00, 100.0]. The corresponding FIS error [−1.0, 1.0] between the ground truth image and the model predicted image is then calculated from:

$$FIS error={(FI\%}_{Pred}-{FI\%}_{Ground Truth})/{FI\%}_{Ground Truth}.$$

At each voxel along the complete fissure the label accuracies were computed as:

$${ACC}_{FIS}\%= \# correctly\, labeled\, voxels/\# voxels\, complete\, fissure*100.$$

In this way ${ACC}_{FIS}\%$ falls within the range [0.0,100.0]. Area under the ROC curve was also computed for classifying fissures as “complete” (≥ 90% complete; as the positive class) versus “partial” (≥ 10% complete) and “missing” (< 10% complete) as defined in^11,12^. Finally, IntegrityNet’s performance on different levels of COPD severity was assessed to evaluate the model’s robustness to disease status.

In order to test the reproducibility of the fissure integrity predictions, subjects’ images across time (one-year, two-year, and five-year follow-up) were processed independently and their $FI\%$ were compared.

**Figure 4:**
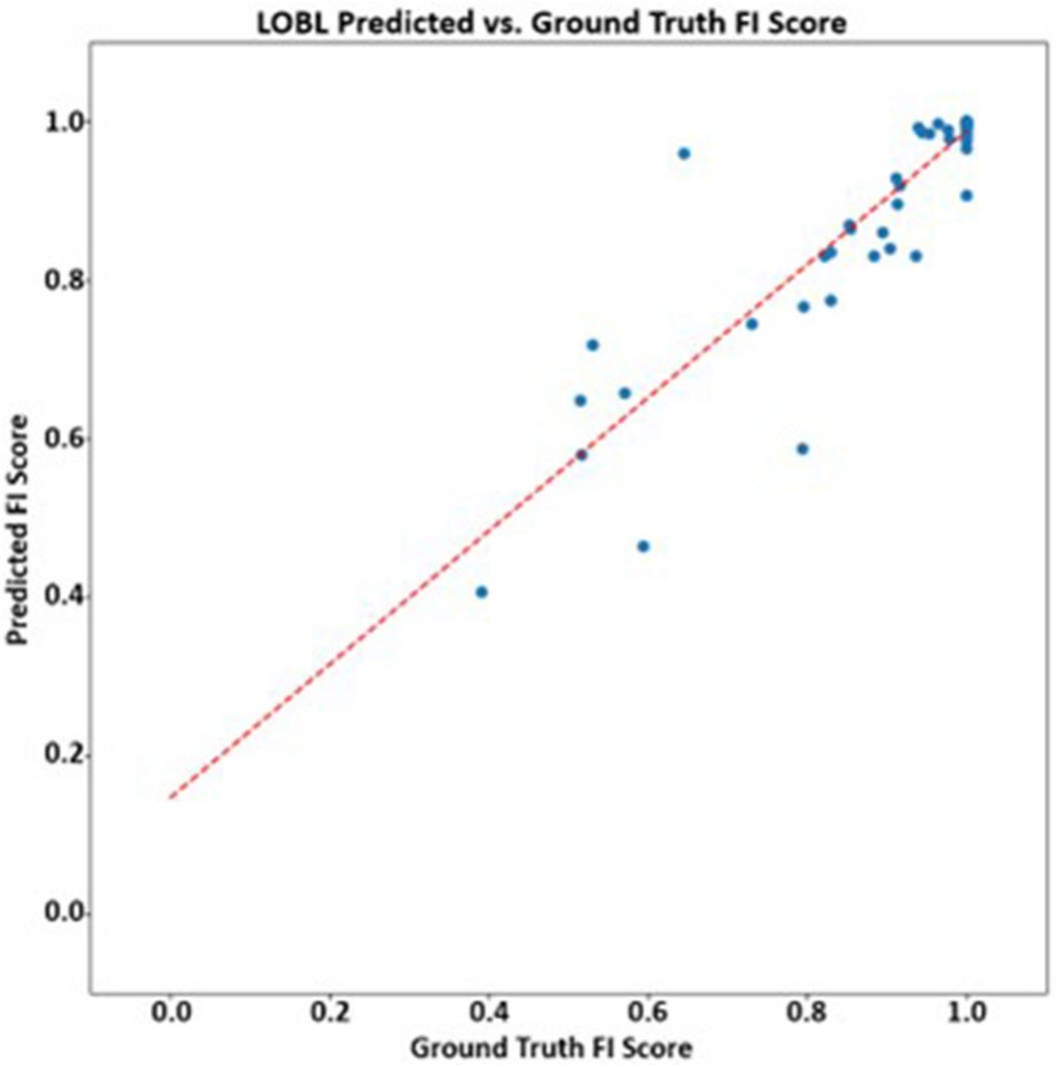
Left oblique fissure predicted fissure integrity score compared to ground truth. Red dashed line represents linear trendline.

**Figure 5:**
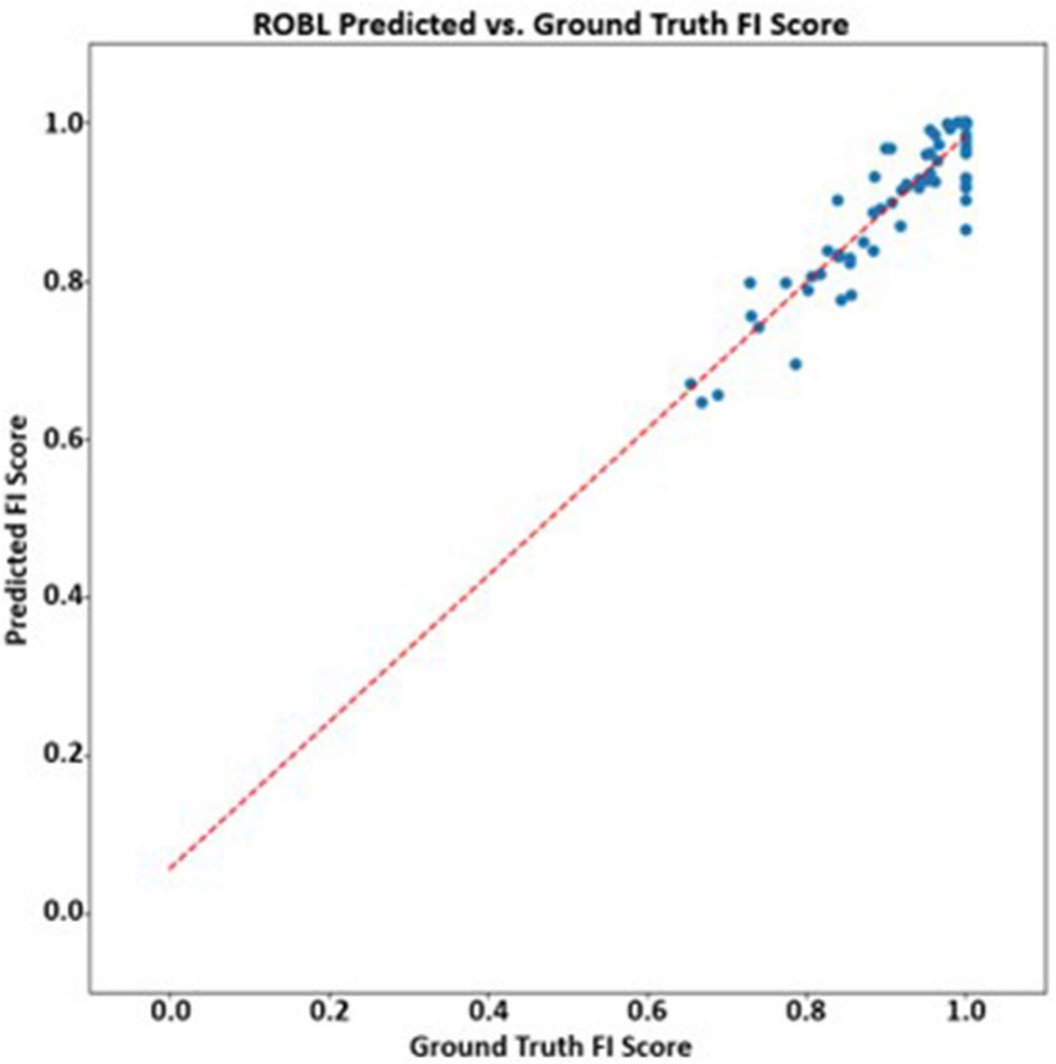
Right oblique fissure predicted fissure integrity score compared to ground truth. Red dashed line represents linear trendline.

**Figure 6:**
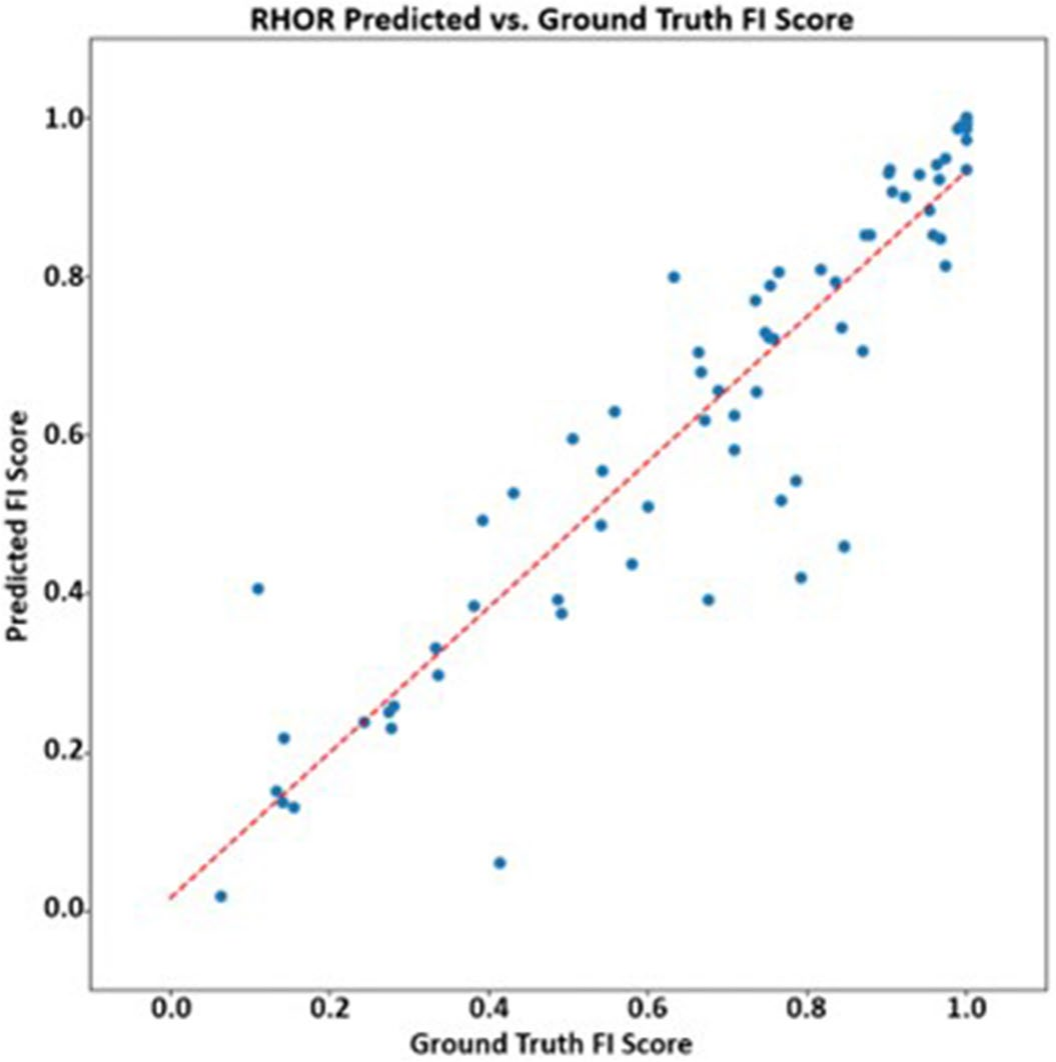
Right horizontal fissure predicted fissure integrity score compared to ground truth. Red dashed line represents linear trendline.

Results from the $FIS error$, ${ACC}_{FIS}\%$, and AUC studies are shown below in Table 2. It can be seen that IntegrityNet correctly classified voxels along the fissure as complete/incomplete over 89% for all three fissures. Figures 4, 5, 6 show predicted and ground truth FI scores for each fissure. R^2^ values for the distributions were equal to 0.84 for both left and right oblique fissures and 0.85 for the right horizontal fissure. Comparison of results across COPD severity groups are given in Table 3 and show similar accuracies for each fissure across all severity groups analyzed in this study. Reproducibility results are shown in Table 4. The average absolute difference in assessed FI% for each fissure and time point comparison did not exceed 4%.

**Table 2:** FIS error, ACC_FIS_%, and AUC results from the eightfold cross validation study (mean $\pm$ standard deviation).

Fissure	$FIS error$	${ACC}_{FIS}\%$	AUC
LOBL	0.045 ± 0.066	95.828 ± 4.882	0.973 ± 0.047
ROBL	0.029 ± 0.026	96.061 ± 2.618	0.914 ± 0.095
RHOR	0.163 ± 0.223	89.810 ± 8.583	0.851 ± 0.114

**Table 3:** Study of model robustness against disease severity (mean $\pm$ standard deviation).

COPD severity	Left oblique ACC_FIS_%	Right oblique ACC_FIS_%	Right horizontal ACC_FIS_%
Normal	97.155 ± 3.829	98.116 ± 2.089	94.170 ± 4.163
GOLD 0	98.411 ± 2.704	98.897 ± 1.229	90.237 ± 10.276
GOLD 1	97.476 ± 2.140	97.853 ± 3.681	95.706 ± 2.485
GOLD 2	96.985 ± 2.841	97.875 ± 1.945	94.736 ± 4.244
GOLD 3	94.600 ± 7.653	97.768 ± 1.240	94.126 ± 4.601
GOLD 4	96.582 ± 3.073	98.423 ± 0.979	94.975 ± 3.837

**Table 4:** Reproducibility study. Model predictions compared across study time points by absolute percent error between FI% measurements. (mean $\pm$ standard deviation).

Fissure	Absolute percent difference		
	Baseline versus 1-year	1-year versus 5-year	Baseline versus 5-year
LOBL	1.372 ± 2.072	1.521 ± 2.380	1.133 ± 1.846
ROBL	1.074 ± 2.368	1.056 ± 1.769	0.805 ± 0.982
RHOR	3.945 ± 6.272	2.412 ± 2.241	2.791 ± 2.981

## Discussion

We have shown that IntegrityNet is able to accurately assess fissure integrity compared to manually annotated fissures. Our method shows results that are comparable to methods reported in the past^10–13^. After training, the average processing time was around 25 s per case (2.5 $\pm$ 1.6 s preprocessing, 8.0 $\pm$ 2.1 s model prediction, 12.2 $\pm$ 0.4 s postprocessing), greatly reducing the analysis time compared to visual assessment which can take anywhere from several minutes to an hour to perform if annotations are required. We have also shown that the model is robust to differences in GOLD stages of COPD severity as the results are consistent across all groups. Model predictions were also shown to be consistent across images taken from the same subject at different time points within SPIROMICS. Widescale deployment of our method across the imaging dataset used in this study is possible, however further validation of the robustness to different study imaging protocols (e.g., slice thickness, CT dose, CT kernel, etc.) is necessary before deployment on other cohorts.

**Figure 7:**
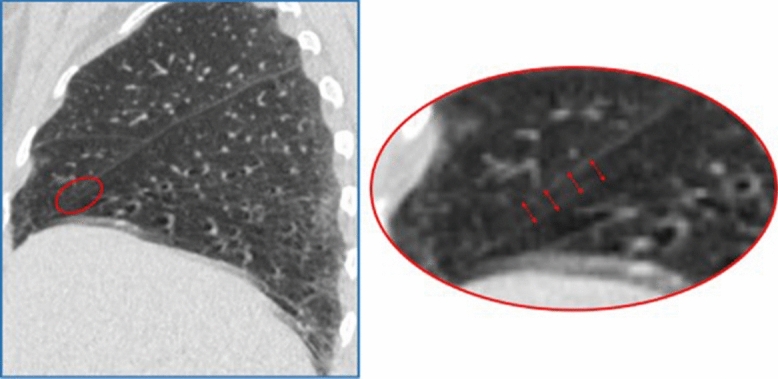
Example of a motion induced blurring artifact in a CT image.

One important limitation of IntegrityNet, as well as other imaging-based fissure integrity assessment tools, is that the method is trained and validated only on the radiographic appearance of the fissure surface. There are many factors outside of fissure completeness that can impact how the fissure is visualized in the CT image. Image motion artifacts, metal artifacts, and pathologies resembling the fissure can all lead to incorrect classification of the fissure surface. An example motion artifact obscuring the appearance of the fissure can be seen in Fig. 7.

Another limitation of IntegrityNet is that it relies on fissure segmentation success. If abnormal anatomy is present and the segmentation method cannot segment the anomalous features properly, IntegrityNet will not be able to handle this case. Since this study only used cases with normal three-fissure anatomy and successful fissure segmentations, our results are likely biased toward the positive.

Additional work is needed to examine robustness to variations in image acquisition protocols, anatomic variants, and to pulmonary pathologies beyond COPD. Although only inspiratory CT scans were used in this work, FissureNet has been validated on expiratory images as well^18^. Assessment of model performance on expiratory images using the network trained on the inspiratory images will illuminate its ability to be readily deployed on exhale scans or the need for a separately trained model for use on expiratory images.

There is a range of fissure completeness within the cohort used for this study. A breakdown of the average manually-assessed FI% for each fissure is given in Table 5. Similar to distributions discussed by studies examining fissure integrity trends in cadavers^6–9^ each fissure is seen to be fully intact less than 80% of the time. The right horizontal fissure was observed to have the most variability in fissure completeness, again consistent with reports from prior study of fissure integrity trends^6–9^.

**Table 5:** Average Fissure Integrity within the Dataset.

Fissure	Average ground truth fissure integrity
Left oblique	89.818 ± 16.496%
Right oblique	91.850 ± 9.253%
Right Horizontal	67.621 ± 28.487%

We have developed a novel deep-learning based fissure integrity assessment method that is suitable for deployment on large collections of CT image data, such as those collected in clinical trials. Our method builds off a state-of-the-art fissure segmentation method and utilizes global information by processing the whole lung CT image data rather than local image patches, in contrast to other methods. By applying our method to a large, well-characterized cohort of subjects with and without smoking history who have COPD, we have validated our model’s performance across a range of disease levels. We have also reproduced the results of prior non-automated studies of fissure integrity variation by showing that fissure completeness in our cohort varied greatly between subjects using our automatic method (Table 5)^6–9^. Beyond the publicly available CT data used in this study, we are releasing our fissure segmentations and ground truth fissure integrity annotations so that others can reproduce our work and develop fissure integrity analysis tools of their own. Automated fissure integrity assessment methods will allow future study of fissure integrity’s relation to disease severity and progression, pulmonary biomechanics, and other demographic variables of interest that have yet to be well defined.

### Supplementary Information

Supplementary Information.

## Data Availability

The CT image data, fissure segmentations, and fissure incompleteness annotations used in this study are available from the SPIROMICS data coordinating center. See https://www.spiromics.org/ for instructions on how to request access to the data.

## References

[1] Eberhardt R, Daniela G, Herth FJF, Schuhmann M (2015). Endoscopic bronchial valve treatment: Patient selection and special considerations. Int. J. Chron. Obstruct. Pulmon. Dis..

[2] Koster TD, Slebos DJ (2016). The fissure: Interlobar collateral ventilation and implications for endoscopic therapy in emphysema. Int. J. Chron. Obstruct. Pulmon. Dis..

[3] Koster TD (2016). Predicting lung volume reduction after endobronchial valve therapy is maximized using a combination of diagnostic tools. Respiration.

[4] Sciurba FC (2010). A randomized study of endobronchial valves for advanced emphysema. N. Engl. J. Med..

[5] Galloy AE, Amelon RE, Reinhardt JM, Raghavan ML (2022). Contact mechanics model of lung lobar sliding. Appl. Eng. Sci..

[6] Hermanova Z, Ctvrtlik F, Herman M (2014). Incomplete and accessory fissures of the lung evaluated by high-resolution computed tomography. Eur. J. Radiol..

[7] Joshi A (2022). Variations in pulmonary fissure: A source of collateral ventilation and its clinical significance. Cureus.

[8] Sudikshya KC, Shrestha P, Shah AK, Jha AK (2018). Variations in human pulmonary fissures and lobes: A study conducted in Nepalese cadavers. Anat. Cell Biol..

[9] Mutua V (2021). Variations in the human pulmonary fissures and lobes: A cadaveric study. Open Access Lib. J..

[10] van Rikxoort EM (2012). A method for the automatic quantification of the completeness of pulmonary fissures: Evaluation in a database of subjects with severe emphysema. Eur. J. Radiol..

[11] Pu J (2010). Computerized assessment of pulmonary fissure integrity using high resolution CT. Med. Phys..

[12] Ross JC (2020). An open-source framework for pulmonary fissure completeness assessment. Comput. Med. Imaging Graph..

[13] Tada, D. K., et al. 3D patch-based CNN for fissure segmentation on CT images to quantitatively assess fissure integrity and evaluate emphysema patients for endobronchial valve treatment. *Proceedings of the SPIE*, vol. 12465 (2023).

[14] Couper D (2014). Design of the Subpopulations and Intermediate Outcomes in COPD Study (SPIROMICS). Thorax.

[15] Sieren JP (2016). SPIROMICS protocol for multicenter quantitative computed tomography to phenotype the lungs. Am. J. Respirat. Crit. Care Med..

[16] Vestbo J (2013). Global strategy for the diagnosis, management, and prevention of chronic obstructive pulmonary disease: GOLD executive summary. Am. J. Respirat. Crit. Care Med..

[17] Doel T, Gavaghan DJ, Grau V (2015). Review of automatic pulmonary lobe segmentation methods from CT. Comput. Med. Imaging Graph..

[18] Gerard SE, Patton TJ, Christensen GE, Bayouth JE, Reinhardt JM (2019). FissureNet: A deep learning approach for pulmonary fissure detection in CT images. IEEE Trans. Med. Imaging.

[19] Lassen B (2013). Automatic segmentation of the pulmonary lobes from chest CT scans based on fissures vessels and bronchi. IEEE Trans. Med. Imaging.

[20] van Rikxoort EM, Prokop M, de Hoop B, Viergever MA, Pluim JPW, van Ginneken B (2010). Automatic segmentation of pulmonary lobes robust against incomplete fissures. IEEE Trans. Med. Imag..

[21] Gerard, S. E., Reinhardt, J. M., Pulmonary Lobe Segmentation Using A Sequence of Convolutional Neural Networks For Marginal Learning. *2019 IEEE 16th International Symposium on Biomedical Imaging (ISBI 2019)*, pp. 1207–1211, 2019b, doi: 10.1109/ISBI.2019.8759212

[22] 3D Slicer. https://www.slicer.org/

[23] Ronneberger, O., Fischer, P., Brox, T. U-net: Convolutional networks for biomedical image segmentation. arXiv:1505.04597 (2015).

[24] Oktay, O., et al. Attention U-Net: Learning Where to Look for the Pancreas. arXiv:1804.03999 (2018).

[25] Chollet, F., et al. Keras. GitHub. https://github.com/fchollet/keras

[26] Kingma, D. P., Ba, J. Adam: A method for stochastic optimization. arXiv preprint, arXiv:1412.6980 (2014)

[27] Salehi SSM, Erdogmus D, Gholipour A (2017). Tversky loss function for image segmentation using 3D fully convolutional deep networks. Mach. Learn. Med. Imag..

